# 2239: Mobile enhancement of motivation in schizophrenia: A pilot trial of a personalized text message intervention for motivation deficits

**DOI:** 10.1017/cts.2017.147

**Published:** 2018-05-10

**Authors:** Lauren Luther, Bryan P. McCormick, Christopher C. Lapish, Michelle P. Salyers

**Affiliations:** Indiana University School of Medicine, Indianapolis, IN, USA

## Abstract

OBJECTIVES/SPECIFIC AIMS: Motivation deficits are one of the strongest determinants of poor functional outcomes in people with schizophrenia. Mobile interventions are a promising approach to improving these deficits, as they can provide frequent cues and reinforcements that support goal-directed behavior. The objective of this study is to describe the intervention protocol and initial effectiveness of a personalized mobile text message intervention, Mobile Enhancement of Motivation in Schizophrenia (MEMS). METHODS/STUDY POPULATION: This pilot study will examine the effects of MEMS compared with a control group using a randomized design. Up to 40 outpatients with a schizophrenia-spectrum disorder will be recruited. All participants will set individualized recovery goals to complete over an 8-week period; those randomized to receive MEMS will also receive 3 sets of personalized, interactive text messages each weekday to reinforce and cue goal completion. Before and after the 8-week period, participants in both groups will complete validated measures of motivation, quality of life, and functioning. Both groups will also report their goal attainment after 8 weeks. RESULTS/ANTICIPATED RESULTS: It is anticipated that those in the MEMS group will demonstrate greater goal attainment and improvements in motivation, quality of life, and functioning compared with the control group. DISCUSSION/SIGNIFICANCE OF IMPACT: This project will test the initial effectiveness of a novel intervention for improving one of the most debilitating aspects of schizophrenia.

